# Oxytocin/Oxytocin Receptor Signalling in the Gastrointestinal System: Mechanisms and Therapeutic Potential

**DOI:** 10.3390/ijms252010935

**Published:** 2024-10-11

**Authors:** Huiping Liu, Gangqiang Yang, Hongbo Wang

**Affiliations:** School of Pharmacy, Yantai University, Yantai 264005, China; oceanygq@ytu.edu.cn (G.Y.); hongbowang@ytu.edu.cn (H.W.)

**Keywords:** oxytocin, oxytocin receptor, gastrointestinal tract, gut motility, inflammation, gastrointestinal diseases

## Abstract

The neuropeptide hormone oxytocin (OT) is involved in various physiological and pathological processes via the oxytocin receptor (OTR). While OT is most widely known as a reproductive system hormone and a nervous system neurotransmitter, the OT/OTR system has gradually gained much attention for its role in the gastrointestinal (GI) system, such as the GI motility, secretion, and bowel inflammatory reactions. Its importance in GI cancers has also been reported in the past few decades. The promising clinical observations have revealed OT’s anti-nociceptive effect, protective effect over gut injury, and the potential of using microbiota to naturally increase endogenous OT levels, which shed a light on the management of GI disorders with lower side effects. However, no current comprehensive review is available on the actions of OT/OTR in the GI tract. This review aims to present the lesser-known role of the OT/OTR system in the GI tract, and the most recent findings are discussed regarding the distribution and functional role of OTR signalling in regulating (patho)physiological functions of the GI tract. Special emphasis is placed on its therapeutic potential for clinical management of GI disorders, such as GI pain, inflammatory bowel disease (IBD), and irritable bowel syndrome (IBS). The recent characterisation of the OTR’s crystal structure has advanced research for designing and identifying new OTR-specific molecules. Future in-depth basic and clinical research is needed to further elucidate the involvement and detailed mechanism of OT/OTR in GI disorders, and the development of OTR-specific ligands.

## 1. Introduction

The neuropeptide and hormone oxytocin (OT) has been considered as a key regulator of diverse central and peripheral biological functions via the oxytocin receptor (OTR), which is a class A G protein-coupled receptor (GPCR) [1,2]. The OT/OTR system was initially studied for its contribution to the stimulation of uterine smooth muscle contraction during parturition and milk ejection in breastfeeding. OT acts as a neurotransmitter and modulates pro-social behavioural effects such as sexual and maternal care, pair bonding, trust, and empathy in the central nervous system (CNS) [3,4,5].

**Figure 1:**
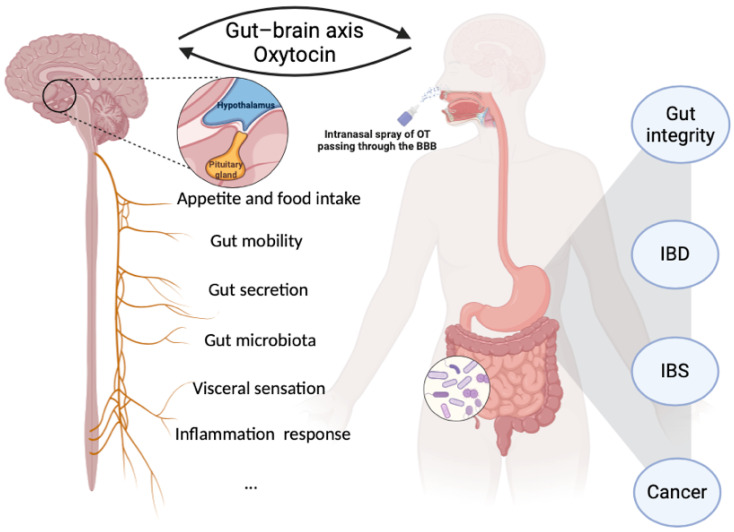
Oxytocinergic signalling is closely involved in the regulation of gastrointestinal physiological function through the gut–brain axis. In the brain, OT is mainly produced in hypothalamic OT neurons in the supraoptic nucleus and paraventricular nucleus and is released from the posterior pituitary gland. Peripheral OT can barely cross the blood–brain barrier (BBB), while intranasally administered OT can pass through the BBB via olfactory and trigeminal nerve fibres. IBD, inflammatory bowel disease; IBS, irritable bowel syndrome. Created with Biorender.com.

The CNS modulates the gastrointestinal (GI) tract via the sympathetic and parasympathetic branches of the autonomic nervous system and the hypothalamic–pituitary axis. OT can be produced in the CNS to exert endocrine functions in response to stimuli or produced locally in peripheral tissue to exert paracrine and autocrine functions [6]. Converging evidence points towards a broader distribution and more roles for the OT/OTR system in various tissues regulating (patho)physiological functions, such as cardiovascular function [7], skin homoeostasis [8], and metabolism regulation [9]. In the past few decades, reports have suggested multiple aspects of the OT/OTR system contributing to the regulation of GI physiology, including motility, inflammation, macromolecular permeability, mucosal maintenance, colonic visceral perception, and gut microbiota [10,11,12,13,14,15]. OT has shown therapeutic potential in animal models of GI diseases and some human studies. Oxytocinergic signalling could be involved in the regulation of GI physiological function through the gut–brain axis (Figure 1). Highlighting the potential role of the OT/OTR system in digestive health adds to evidence of the importance of the gut–brain axis. In this review, we discuss the current studies and knowledge of OT/OTR in the GI tract and attempt to unravel the unresolved issues and promising directions for future investigation.

## 2. Oxytocin/Oxytocin Receptor Presence and Distribution in the Gastrointestinal Tract

OT is a cyclic nonapeptide (CYIQNCPLG) with a single disulphide bond and a C-terminal amide, encoded by the OT gene (*Oxt*, Gene ID: 5020) located at chromosome 3p25.3 [16]. OT is mainly produced in hypothalamic OT neurons in the supraoptic nucleus (SON), paraventricular nucleus (PVN), accessory nuclei between the SON and PVN, and extrahypothalamic regions in the CNS [17,18]. Brain OT plays a crucial role in modulating GI functions [19], but the origin of OT is not restricted to the CNS. The vagus nerve provides bidirectional communication between peripheral organs (heart, lungs, pancreas, liver, stomach, and intestines) and the CNS [20]. OT has been identified in the dorsal vagal complex (DVC) [21], which has a significant role in controlling subdiaphragmatic visceral organs. OT cells are also present in peripheral sites including the GI tract [22,23]. Immunohistochemistry has confirmed that enteric OT protein expression is restricted to neuronal elements in the human GI tract [22], and in intrinsic sensory and secretomotor neurons in the enteric nervous system of guinea pig [23]. However, a recent study showed that OT can also be efficiently produced in the intestinal epithelium [24]. These data indicate that OT can contribute its role in GI (patho)physiology both centrally and locally.

OT exerts its effects through binding to the OTR. For a functional OT/OTR system, stimulus-dependent expression and release of OT have to be balanced with a fine-tuned regulation of local OTR expression [5]. In humans, a single type of OTR is expressed which is encoded by the OTR gene (*oxtr*) made up of three introns and four exons located at 3p25.3 (Gene ID: 5021) [1,18]. OT and OTR are expressed throughout the GI tract in the human body [22,25]. Enteric OTR protein expression is not confined to neuronal cells in rats; OTR was observed using immunohistochemistry to be located in the myenteric plexus of the colon [26], and OTR presence was also observed in crypt cells, especially at crypt–villus junctions in the gut [27]. Crypt cells are a contiguous pocket of epithelial cells at the base of the villus with rapid and continuous proliferation abilities, supporting the self-renewal and cell differentiation of the intestinal epithelium. The OTR’s presence in crypt cells is consistent with the distribution of the proliferation-regulating proteins controlled by various pathways [28], and this has been supported by the observation of shorter villi and crypts in OTRKO mice compared to wild type mice [13]. Enteric OT and OTR expression in both the enteric nervous system (ENS) and the epithelium is developmentally regulated through adulthood, peaking at postnatal day 7 in rats. Strong OTR immunoreactivity in villus enterocytes lasted until postnatal day 17 (i.e., until weaning and crypt formation) [27]. Considering the fact of the presence of OT in milk [29], it is convincible that OT may play critical roles in the neonatal stomach and intestine and that OT/OTR signalling contributes to the maturation of the bowel by modulating the migration of newly generated enterocytes or in their function in the new-born gut. OTR expression on the epithelial cells was stably maintained through adulthood once acquired, implying that the function of enteric OT/OTR signalling is unlikely to be restricted to development but through the lifetime of the adult [27]. The adult expression pattern of the OTR is suggestive of the role of OT in modulating secretion and/or epithelial renewal processes (the crypt is the site of enterogenesis in the adult). Besides the enteric system, OTRs are also present in the smooth muscles of the gastric body in rats, which might mediate the excitatory effect of OT [30].

The distribution of OT and OTR in the same tissue could lend support to speculations about the existence of an autocrine and/or paracrine loop in the GI tract. The neonatal and adult expression patterns of the OT/OTR are strong foundations to presume an essential role of OT/OTR signalling in GI physiology and pathology. For the successful clinical application of OT/OTR-based therapy in the GI system, one approach is to enhance our understanding of OTR signalling in gut physiology and pathophysiology.

## 3. Oxytocin/Oxytocin Receptor and Gastrointestinal Physiology

The principal physiological functions of the GI tract are to digest and absorb ingested nutrients and to excrete the waste products of digestion, which is coordinated by a series of complex events such as neural control, intestinal microenvironment, extrinsic intervention, and so on [31]. Multiple neurotransmitters can affect GI functions, including serotonin, acetylcholine, norepinephrine, epinephrine, dopamine, ghrelin, orexin, neuropeptide Y (NPY), alpha-melanocyte-stimulating hormone (alpha-MSH), and OT [32,33]. The OT/OTR system could be a crucial driver for GI health, especially considering the concept of the gut–brain axis in the pathophysiology of both neuropsychiatric and GI functions. The OT/OTR system is involved with several physiological functions in the GI tract, such as modulation of GI motility, gastric secretion, mucosal integrity, and colonic permeability [13], which are discussed in this section.

### 3.1. Food Intake

There is widespread expression of OTRs in CNS sites that are linked to the control of food intake. OT plays a critical role in eating disorders, with potential therapeutic perspectives [34]. OT may act as a satiety hormone, but its exact role and mechanism in regulating food intake is not fully understood. Food intake stimulates oxytocinergic neurons in response to physiological cues such as gastric distension and increased plasma osmolality, suggesting an anorexigenic effect of OT [35]. Increasing evidence has demonstrated that central OT signalling decreases food intake and caloric consumption, which may further affect gut microbiota and gut functions [36,37]. It is hard for the peripheral applied OT to pass the blood–brain barrier to work within the brain. Intranasal spray application of OT can affect the processing of emotional stimuli and exert anti-anxiety effects, most likely by modulating amygdala activity [38]. Intranasal administration is now the commonly used experimental protocol for studying the role of central OT signalling (Figure 1), although there is still debate on the amount or even whether intranasally administered OT enters the brain.

In humans, OT appears to restrain food intake by enhancing the activity of fronto-cortical brain areas that establish cognitive control and process reward, thereby overriding the hedonic drive to eat [39]. Multiple human studies are currently under investigation to assess the potential of OT as a therapeutic agent in the pathophysiology of obesity and metabolic syndrome, which is closely linked to eating behaviour. Interestingly, OT administration on food intake is not limited to its anorexigenic effect; it may have contrary effects according to specific (patho)physiological conditions, as well as certain behavioural and social contexts [40]. For example, neither OT nor AT significantly affect satiety in healthy subjects [41], while early research has suggested that dysfunction of OT-ergic mechanisms is associated with anorexia nervosa (AN), with specific patterns including lower circulating levels of OT at fasting and after stimulation, lower nocturnal levels of OT, and higher peripheral OT concentration after meal ingestion [42]. However, a recent phase II clinical trial showed no significant differences between intranasal-OT and placebo groups on the outcomes of interest in AN patients [43]. The effects and mechanisms of OT on food intake control and eating behaviour have been reviewed in more detail elsewhere [9,34,36].

Many patients diagnosed with an eating disorder also receive a diagnosis for at least one more psychiatric disorder, such as depression, anxiety disorder, schizophrenia, or systemic developmental disorders [38]. Special studies with large sample sizes are surely necessary to verify more precisely the better mechanisms and therapeutic role of OT in eating disorders, so that more patients could benefit from OT-based therapies.

### 3.2. Gastrointestinal Motility

Conflicting results between animals and humans have been reported regarding GI motility regulated by the OT/OTR system. The effects of OT/OTR on GI motility may depend on species, subject gender, the site within the GI tract, the concentration of OT, the expression level of OTRs, or even the stimulation the subjects suffered (Table 1).

In the human body, reduced postprandial secretion of OT was observed in gastroparesis patients with delayed gastric emptying [44]. OT promotes GI motility and is involved in postprandial release [12,45]. The prokinetic effect of OT on the gut is assumed to be similar to that in uterine myometrium and mammary myoepithelial cells related to the intracellular release of Ca^2+^, which leads to muscle contraction via myosin light kinase activity [18,44]. Another study showed that the oxytocin/vasopressin receptor antagonist atosiban (AT) delayed the gastric emptying of a semisolid meal [41], while in the same study, infusion of the actual pharmacological dose of OT (40 mU/min) did not affect the gastric emptying. Hence, the dose and precise mechanism of OT for the action in the human stomach might be related to the differential physical environment, which still needs to be further illuminated.

In rats, the in vitro data showed that OT induced a prompt contraction by directly binding to OTRs on cell membranes [30], while multiple in vivo studies showed that OT reduced gastric motility [46,47,48,49,50]. Interestingly, in one study it was shown that OT did not influence colon motility if the rats under study were at the diestrus period, but it significantly increased the colon pressure if the rats were at other stages [26]. Both acute stress and long-term chronic heterotypic stress can accelerate colonic transit partially through OT/OTR signalling [26,46,47], which is oestrogen dependent [26,47]. The published data indicate that the effects of OT on GI motility may depend on the sex of the subjects and the site of the GI tract. Another study showed that OT can induce an early transient decrease in and a subsequent excitation of gastric contraction. Interestingly, the transient inhibition may be due to the secretion of cholecystokinin (CCK) [30]. Both endogenous and exogenous CCK stimulation by consuming a fatty meal or through the injection of CCK can lead to OT secretion in women [51]; therefore, it can be speculated that the effects of OT may be mediated indirectly by CCK signals. Another study showed that microinjection of OT in rats decreased gastric tone and motility in a dose-dependent manner, which is related to the activation of the non-adrenergic non-cholinergic (NANC) pathway and modulation of gamma-aminobutyric acidergic (GABAergic) transmission between the nucleus of the tractus solitarius (NTS) and the dorsal motor nucleus of the vagus (DMN) [50].

In mice, the opposite effects of stress on gastric motility were observed compared to rats. Acute and chronic repeated restraint stress can inhibit gastric motility and delay gastric emptying, which is correlated with the upregulation of central OT; furthermore, intracerebroventricular injection of OT can restore the impaired gastric motility [52]. In a recent study, OT pretreatment was observed to ameliorate the inhibition of GI motility caused by vincristine [53]. The OT/OTR signalling system in GI function was further studied both in wild-type and OTR-knockout (OTRKO) mice [13]. OT injection prolongs GI transit time (GITT) in both BALB/c and C57BL/6 wild-type mice, while OTRKO in C57BL/6 accelerates transit; OTR knock-out also caused deficient cell proliferation in villi and crypts. Interestingly, OT slowed both gastric emptying and small bowel transit in BALB/c mice, but only delayed gastric emptying in C57BL/6 mice without significant influence on small bowel transit. Inhibition effects of OT on gastric motility were also observed in other animal models; for example, OT inhibits colon contraction in rabbits, which is partly relevant with the N receptor [54,55] and gastric antrum in guinea pigs resulting from the activation of Ca^2+^-sensitive K^+^ conductivity [56].

Therefore, in the human GI tract the effects of OT appear to be direct, while in animal models, the effects of OT on GI motor function are mediated vagally [57] or indirectly by other factors. However, many animal experiments are performed under anaesthesia and involve acute surgical preparation, and in some animal studies the OT administration is at supraphysiological levels, at which OT can also bind with vasopressin receptors (VPRs); for these reasons, it might be difficult and inaccurate to understand the physiological/pathological effects and mechanisms of the OTR system only using animal models in terms of normal physiology. Further studies are warranted for a better understanding of the role of the OT/OTR system in GI motility regulation.

**Table 1:** Summary of oxytocin effects on gastrointestinal motility.

Species	Treatments	Effects on Gastric Motility	Refs.
Human	OT, 5–20 U in 500 mL saline, i.v. infusion in 4 h for 3 days	OT accelerated gastric motility in patients with duodenal ulcer complicated by pyloric stenosis.	[45]
	OT, 20 U in 500 mL saline, i.v. infusion, 0.33 U/min	OT increased the basal gastric emptying rate in healthy subjects.	[12]
	OT, 5 U in 250 mL saline, i.v. infusion, 40 mU/min AT, intravenous bolus dose of 6.75 mg AT immediately followed by a continuous i.v. infusion of 6.75 mg AT in 250 mL saline	OT had no effects, and AT reduced gastric emptying rate in healthy volunteers.	[41]
Rats	OT, in vitro, 10^−9^–10^−6^ M; in vivo, 1.5 μg/kg, i.v. injection	In vitro, OT increased the contraction of the muscle strips of gastric body, antrum, and pyloric sphincter, and decreased the average cell length of isolated smooth muscle cells. In vivo, systemic administration of OT induced an early transient decrease and a subsequent increase in intragastric pressure, and the transient inhibition could be abolished by pre-treatment with the CCK1 antagonist, devazepide.	[30]
	OT, 0.5 μg ICV injection prior to restraint stress for 90 min	Acute stressor and chronic heterotypic stress induced accelerated colonic transit, which can be attenuated by ICV injection of OT.	[46]
	OT, 0.2, 0.4, or 0.8 mg/kg, intraperitoneal injection	OT inhibited gastric emptying and gastrointestinal transit in female rats	[48]
	OT, injection into the DMN, 4 pmoles in 2 nanoliters ACSF	OT injection into the DMN caused a reduction in contractile activity. The OT antagonist ETOV injection into the DMN prior to hypothalamus PVN stimulation caused an immediate increase in gastric motility and augmented the motility increase to subsequent PVN_mp_ stimulation.	[49]
	OT, 10 μg ICV injection	OT administration and electrical stimulation of the PVN reduced gastric motility in unanesthetized freely moving rats, which was blocked by i.c.v. administration of an OT antagonist.	[58]
	OT 50–300 pmoles, DVC microinjection	OT decreased gastric tone in a dose-dependent manner, which can be enhanced following bethanechol and reduced by L-NAME administration, suggesting a nitrergic mechanism of gastroinhibition. The group II mGluR antagonist EGLU injected in the DMV induced a gastroinhibition shifted the gastric effects of OT to a cholinergic-mediated mechanism via modulation of GABAergic transmission between the NTS and DMN	[50]
	OT, low, 0.01–0.3 μM; high, 1 or 3 μM	Low concentrations of OT (0.01–0.3 μM) failed to elicit any effect on the contractions of colonic smooth muscle strips. High concentration of OT (1 or 3 μM) decreased the ratio of the contractile force of colonic smooth muscle strips. Ovariectomy decreased the OT-induced inhibition of colonic contractility in intragastric cold water stressed rats, indicating the effect of OT is oestrogen dependent.	[47]
	OT, 1.5 μg/kg i.v. in vivo, or 10^7^ M in vitro	OT did not influence the colon motility if the rats were at diestrus period but significantly increased the colon pressure if the rats were at the other stages. Pretreatment of oestradiol (4–100 μg/kg s.c.) dose-dependently increased the excitatory effect of OT on colon motility both in vivo and in vitro and upregulated OTR expression in colon.	[26]
Mouse	OTR deletion OT, 100 μg/kg subcutaneous injection	OT slowed gastric emptying both in C57BL/6 and in BALB/c mice. OT slowed small intestinal transit in BALB/c mice but not in C57BL/6 mice. OT slowed while OTRKO accelerates GI transit time in C57BL/6 mice.	[13]
	OT 0.1 and 0.5 μg, ICV injection	OT restored the delayed gastric emptying and motility induced by acute stress.	[52]
	OT 0.1 mg/kg/d, i.p., 1 h before VCR injection	OT pretreatment ameliorated the inhibition of GI motility and the injury of myenteric neurons caused by vincristine.	[53]
Rabbit	OT, 1–10 U/L in vitro	OT inhibited the motility of proximal colon.	[54]
	OT, 1, 10, 100 nM in vitro	OT inhibits the contractile motility of the distal colon, which was blocked by the OTR antagonist AT. Mg^2+^ and ovarian steroids oestradiol (0.1 μM) or progesterone (0.1 μM) increased the OT-induced response.	[55]
Guinea pig	OT, 10^−12^–10^−8^ M in vitro	OT suppressed in a dose-dependent manner, the tetrodotoxin- and atropine-resistant spontaneous phasic contractions and shifted rightward the dose-response curves of 10^−7^ M charybdotoxin and 10^−3^ M BaCl_2_ in guinea-pig antral smooth muscle.	[56]

OT, oxytocin; AT, atosiban; ETOV, the OT antagonist dE-t_2_Tyr(Et)Orn^8^ Vasotocin; VCR, vincristine; ACSF, artificial cerebrospinal fluid; DMN, dorsal motor nucleus of the vagus; DVC, dorsal vagal complex; ICV, intracerebroventricular; NTS, nucleus of the tractus solitarius; PVN, paraventricular nucleus.

### 3.3. Gastric Secretion

Oxytocinergic signalling may interact with gastric secretion, including gastric acid, ghrelin, and secretin. Early research in rats showed that injections of OT centred on the dorsal motor nucleus of the vagus substantially increased gastric acid secretion, and this effect can be blocked by an OT antagonist (dEt2Tyr(Et)Orn^8^Vasotocin, ETOV) and peripheral injections of atropine, which indicates that the effect of OT on gastric acid secretion is OTR-specific and the effect on the efferent vagal fibres to increase gastric secretion seems to be cholinergic-mediated [59]. However, later studies showed that centrally and peripherally administered OT can reduce gastric acid secretion in guinea pigs and rats [60,61,62,63,64,65]. The effects of OT can be reverted by the OTR antagonist AT in the above studies [61,62,63,64,65]; specifically, OTR gene knockout leads to the loss of OT’s protective effect in the rat model of stress-induced ulcers [64], confirming the involvement of OTR in this course.

Ghrelin is another peptide hormone that can be affected by the OTR system. Ghrelin is the endogenous ligand for the growth hormone secretagogue receptor (GHSR) originally isolated from the rat stomach, and human ghrelin is homologous to rat ghrelin with only two amino acid differences [66]. Ghrelin is secreted in the stomach during fasting and targets the growth hormone secretagogue receptor (GHSR1a) in the hypothalamus and brainstem to exert its orexigenic effect. There are controversial data about effects of OT on ghrelin secretion, too. In healthy men, systemic administration of OT has been shown to reduce basal and lipopolysaccharide-induced ghrelin levels [67]. Meanwhile, in the ghrelin-producing MGN3-1 cell line, OT as well as dopamine were shown to stimulate ghrelin secretion in vitro [68]. In rats, plasma ghrelin concentrations were significantly decreased during pregnancy and lactation, but it was the suckling stimulus itself, not OT that suppressed ghrelin secretion in lactating rats [69]. In pigs, OT administration for the first 14 days of life resulted in greater stomach ghrelin and leptin expression seven days after weaning [70]. The reason for this discrepancy is not clear, and further studies are needed to explore the regulation of ghrelin secretion by OT in vivo.

On the other hand, the classic GI hormone secretin can also affect OT signalling. Intracerebroventricularly injected secretin was shown to activate the oxytocinergic neurons in the PVN and SON in the rat hypothalamus and increase OT mRNA levels [71]. The influence of systemic secretin was mediated through noradrenergic pathways via α1-adrenoceptors [72]. Stimulation of OT secretion in enterocytes by *Limosilactobacillus reuteri* (*L. reuteri*) is dependent on secretin, which is produced in enteroendocrine cells [24].

Patients with gastric secretion-related disorders may benefit from OT treatment, in particular those in whom OT signalling may be abnormal, e.g., individuals with mental disorders. Future animal studies and clinical trials in humans are needed for detailed assessments of the interaction between oxytocinergic signalling and gastric secretion.

### 3.4. Gut Microbiome

The GI tract interfaces with a luminal environment inhabited by a collection of microbial organisms defined as the gut microbiome. The crosstalk between the gut microbiota and the regulation of the CNS is believed to impact mammalian physical and mental health. The gut–brain axis is the bidirectional neurohumoral link and communication system between the gut microbiota and the central nervous system (CNS) [73]. Oxytocinergic signalling is closely involved with the gut microbiota through the “gut–brain axis”, with multiple studies supporting OT as a novel multi-directional “microbiome–gut–brain axis” component [15,74,75].

In a rat model of corticosterone-induced stress, OT administration not only decreased anxiety/depression-like behaviours, but also induced a ‘microbiota composition shift’ [76]; thus, an increase in *Mogibacterium* following OT treatment has the potential to be identified as a biomarker for OT-induced behavioural changes. Recent research showed that OT can impact and is required for human intestinal microbe *Limosilactobacillus reuteri*-mediated wound healing and social behaviour [24]. On the other hand, alterations in the gut microbiome can impact physical health through OT secretion and signalling regulation. OT and the gut microbiome near birth trigger a dramatic interplay of the intestinal epithelia and the enteric nervous system and guide the development of the intestine and gut–brain axis, influencing both the mother and infant [77]. Administering purified *Lactobacillus reuteri* organisms in drinking water induces a significant upregulation of OT in mice by a vagus nerve-mediated pathway, which could be a main mechanism of the wound-healing promoting properties of *Lactobacillus reuteri* [78]. The fundamental discoveries revealing the interactions between OT and the gut microbiome paved the way for further exploration of the mechanisms in human bodies. Importantly, vagotomy in mice abolished the theory of favourable mood regulation by consumption of *Lactobacillus rhamnosus* [79]. These data suggest that the microbes communicate and influence OT circulation involving the gut–brain axis via the vagus nerve.

The interactions between OT and the gut microbiome provide a possibility of using heirloom microbiota to naturally upregulate host endogenous OT levels and potentially overcome the known therapeutic challenges of OT’s short half-life and obscure brain targets. Further research in humans into the pathways of communication between the gut microbiome, emotional behaviour, and the OT system would lay the foundation for the clinical application of these microbial symbionts and OT for GI disorder management.

## 4. Oxytocin/Oxytocin Receptor and Gastrointestinal Pathological Disorders

GI diseases rank among the most prevalent disorders, referring to diseases of the oesophagus, stomach, small intestine, colon, and rectum, such as GI visceral hypersensitivity, gastroparesis, inflammatory bowel disease (IBD), irritable bowel syndrome (IBS), gastric and peptic ulcers, GI cancers, and so on. Many GI disorders are difficult to diagnose, and their symptoms are not effectively managed. Thus, basic research is required to drive the development of novel therapeutics which are urgently needed. OT/OTR is an essential signalling pathway in gut physiology and pathophysiology. OTR represents a promising therapeutic target for the treatment of these pathological conditions since it has clinical relevance to some GI complaints.

### 4.1. Gastrointestinal Pain

OT is widely distributed in the CNS and spinal cord and is closely involved in pain transmission. OT can influence pain perception in multiple animals and cause analgesia for acute or chronic pain in humans, although there are still controversial opinions [80,81,82]. GI pain is a form of visceral pain arising from the GI tract, which is highly prevalent in diseases such as irritable bowel syndrome (IBS), inflammatory bowel diseases (IBD), pancreatitis, and cancers [83]. Emerging evidence supports the potential of targeting OTR for visceral pain and the anti-nociceptive properties of OT.

OTR expression is significantly upregulated in colonic neurons of mice with chronic visceral hypersensitivity, and stable seleno-OT analogues are potently analgesic in this animal model of chronic abdominal pain [14]. Neonatal maternal separation induces visceral hypersensitivity and visceral pain in rats with significantly increased OTR expression. At the same time, exogenous OT (intraperitoneal injection, 1 mg/kg body weight) attenuated stress-induced visceral pain and enteric glial cells activation in rats, mediated by TLR4/MyD88/NF-κB signalling [84]. The specific OTR antagonist L-368899 administered intraperitoneally causes visceral hypersensitivity in mice [85]. In humans, plasma OT concentration is significantly lower in children with recurrent abdominal pain of psychosomatic origin and in children with inflammatory bowel disease compared to controls [86]. OT reduced intractable cancer pain by 88% for 77 min in a terminally ill cancer patient with a diffuse mesothelioma [87], nasal administration of OT reduces abdominal pain and discomfort in patients with chronic idiopathic constipation [88], whereas continuous intravenous OT significantly increases thresholds for visceral perception in patients with IBS, possibly by acting at the level of visceral afferents [89].

The detailed anti-nociceptive mechanism of OT is not yet fully understood. A study showed that OT acts centrally through the opioid system to modulate visceral sensation [90]. As shown in one study, intracisternally administered OT instead of intraperitoneal injected OT increased the threshold volume of colonic distension-induced abdominal withdrawal reflex in conscious rats, and pretreatment with subcutaneous injection of a peripheral and central opioid antagonist (naloxone hydrochloride) blocked OT-induced visceral anti-nociception [90]. For therapy purposes, if the therapeutic effects are further validated in humans, OT and other agonists targeting OTR may represent a relatively safe form of pharmacotherapy for pain management with little potential for addiction. The challenge would be how to extend the short half-life and lack of specificity of OT by new peptide or non-peptide ligand development or drug delivery techniques.

### 4.2. Gastrointestinal Epithelium Injury and Wound Healing

The GI epithelium presents a physical barrier to invasion by microorganisms and injury from insults, such as bacterial infection, chemotherapy, or radiation injury, upon which the epithelium undergoes a wound-healing process. The OT/OTR system is involved in the repair of intestinal epithelium injury by regulating the proliferation of crypt cells, gastric secretion, mucosal permeability, and protection against inflammation.

Centrally administered OT possesses gastric anti-secretory and anti-ulcer activity, while the antagonist AT is pro-ulcerogenic in rats [61]. Reduced gastric acid secretion by centrally administered OT (i.c.v.) might contribute to reducing gastric and duodenal ulcer formation [61]. Peripherally administered OT (s.c.) also revealed significant anti-ulcer activity on different experimentally induced gastric and duodenal ulcers. OT could significantly protect gastric mucosal against injury induced by ischemia-reperfusion, and the OTR was involved. This effect of OT may be mediated through the vagus and sympathetic nerve, and then lead to the reduction of gastric juice output and the depression of gastric acidity [63]. OT treatment showed significant anti-secretory and anti-ulcer activity in pylorus-ligated rats, in histamine induced acute gastric ulcers in guinea pigs, in cysteamine induced duodenal ulcers in rats, and in acetic acid induced chronic gastric ulcer model. And the anti-ulcer and anti-secretory effect was comparable to that of ranitidine though less in intensity [62]. The anti-ulcer activity of OT is considered to be attributed to its anti-secretory effect.

OT treatment ameliorates oxidant gastric injury and improves the gut barrier integrity in rats with thermal trauma by depressing tissue neutrophil infiltration and decreasing the release of inflammatory factors [91]. Prostaglandin E2 (PGE2) is the most abundant gastrointestinal PG which plays an important role in many gut functions including motility and secretion. OT is a potential novel and safe way to combat or prevent chemo-radiotherapy induced intestine injury by evoking a COX-2 dependent pulsatile release of PGE2 by triggering [Ca^2+^]_i_ oscillations in ileum mucosa [92]. Furthermore, OT can also protect enterocytes and other cell types, such as neurons, from stress-related complications during postnatal development [93].

These data provide potential therapeutic use of OT in the treatment of GI injury. GI mucositis is a common and dose-limiting side effect characterized by inflammatory and/or ulcerative lesions in the mucosa of the digestive tract in patients receiving chemotherapy or radiotherapy. OT can act centrally in the brain to reduce colonic hyperpermeability via the vagal cholinergic pathway or cannabinoid 1 receptor signalling [19]. It has been proposed that OT can have a dose-dependent effect on GI mucositis caused by 5-fluorouracil (5-FU) [94].

### 4.3. Irritable Bowel Syndrome and Inflammatory Bowel Disease

Irritable bowel syndrome (IBS) is a functional GI disorder defined using the Rome IV criteria as a combination of abdominal pain, disordered bowel habits, and bloating, which are similar symptoms as Inflammatory bowel disease (IBD). IBD is a group of inflammatory conditions of the colon and small intestine that arises due to the interaction of environmental and genetic factors leading to immune dysfunction and inflammation responses in the intestine, with Crohn’s disease and ulcerative colitis (UC) being the principal types. IBD and IBS are both long-term conditions, while the inflammation of IBD can cause worse symptoms over time, such as bloody stool and inflammation in other body parts. IBS and IBD commonly co-occur, with about 37% of patients diagnosed with quiescent Crohn’s disease and nearly a third of patients with ulcerative colitis in remission also reporting symptoms of IBS, and patients with IBS and IBD also commonly suffer from stress-related symptoms of anxiety and/or depression [95]. Current treatments for intestinal inflammation have a high percentage of failure and lead to immunosuppression [96]. OT possesses anti-secretory and anti-ulcer effects, facilitates wound healing and is involved in the modulation of immune and inflammatory processes by targeting OTR, which provides a new opportunity to improve the clinical management of IBS, IBD and other GI disorders without compromising the immune system.

Alterations in bidirectional gut–brain axis interaction are considered a crucial cause for the pathogenesis of IBS. Intracisternal injection of OT can improve LPS-induced intestinal hyperpermeability and leaky gut in rats, OT might act as a gut–brain signal mediator to improve intestinal barrier function and visceral sensation and induce a protective action against neuroinflammation in the brain, thereby inducing therapeutic effects on IBS [97]. OT is essential to a wide range of stress-related disorders, including IBS. The decreased colonic motility by OT treatment could help to attenuate the symptoms of IBS induced by stress stimulation [47]. In a long-term water avoidance stress IBS rat model with enhanced intestinal motility and visceral hypersensitivity, more GABAergic projections were found in the paraventricular nucleus (PVN) of the hypothalamus, which inhibited the firing rate of neurons and decreased the expression of OT [98].

Targeting OTR have promising therapeutic potential for IBD especially because the OT/OTR system plays an essential role in both inflammation response and the neuroendocrine-immune network [99,100]. OTR expression levels are significantly lower in colon samples from patients with colitis and patients with colorectal cancer [101]. OTR deletion increased inflammation severity in C57BL/6 mice while OT administration decreased TNF-α and CCR5 levels [13]. OTR deficiency in the myeloid could promote colitis model induction by dextran sulfate sodium (DSS) intervention [102]. The OT levels are also abnormal in the serum or colon tissue of dextran sulfate sodium-induced colitis mice or the plasma of ulcerative colitis patients [103]. Mechanism investigation revealed that OTR depletion in intestinal epithelial cells impairs the inner mucus of the colon epithelium and promotes colorectal cancer progress depending on inflammation, which can be attenuated by OT treatment [101]. OT can function as an anti-inflammatory, antioxidant, and immunomodulatory agent to ameliorate inflammation and attenuate the severity of IBD and sepsis-induced oxidative damage in the colon and liver by a neutrophil-dependent mechanism [104,105]. The OT/OTR system participates in modulating the dendritic cells [103] or polarization of macrophages to an anti-inflammatory phenotype and alleviates experimental colitis [102]. In addition, OT has promising anti-allergenic effects and attenuates systemic anaphylaxis and intestinal inflammation on experimental food allergy by suppressing epithelial Thymic Stromal Lymphopoietin (TSLP), interleukin-25 (IL-25) and IL-33 production via inhibiting NF-κB signalling and upregulating β-arrestin2 expression [106].

These data open new therapeutic perspectives for the clinical management of IBS and IBD using OT to modulate gut motility, improve gut permeability, regulate inflammatory and immune response, and alleviate visceral pain.

### 4.4. Gastrointestinal Cancers

GI cancers are responsible for a significant proportion of cancer incidence and mortality worldwide. Current conventional therapeutic options have significantly increased patient survival, including cytotoxic chemotherapeutics, radiation, immunotherapy, and surgery. However, there has been little advancement in counteracting the pervasive off-target effects and resistance to these treatments, and patients with end-stage disease usually have limited treatment options and an unfavourable prognosis. These limitations have prompted the identification of novel targets and improved treatment strategies for GI cancer management. Previous report has suggested a link between the OT/OTR system and GI-related cancers, such as oesophageal cancer, gastric cancer, and pancreatic cancer [107].

OTR is significantly overexpressed relative to normal tissue in primary and corresponding metastases of small bowel neuroendocrine tumours (SBNETs), pancreatic neuroendocrine tumours (PNETs) [108], and colon adenocarcinoma [109]. Specifically, OTR showed a trend towards higher relative expression in primary/metastasised gastroenteropancreatic neuroendocrine tumours versus normal tissue compared to the somatostatin receptor type 2 (SSTR2) [110], which is currently a known clinical ideal and successful receptor target for imaging and treatment displaying higher expression in tumour tissues with relatively low expression in the adjacent normal tissue. Moreover, the OT/OTR system is involved in the chemoresistance of GI cancer. Both OT and OTR expression is significantly higher in pancreatic cancer cell lines highly unresponsive to the chemotherapeutic agents [111]. GI toxicity is a common complication of chemotherapy, and OT has shown promising protective effects on vincristine-induced GI dysmotility and enteric neuron injury [53]. On the other hand, the cytotoxicity of 5-FU is negatively correlated with OTR expression in microsatellite stable colorectal cancer cell lines. AT has strengthened the antitumor activity of 5-FU by restoring the zinc-finger transcription factor GATA3 [112]. These findings indicate that OTR could be a promising therapeutic target for GI cancers.

### 4.5. Peptide-Based Oxytocin Receptor Ligands Development—Approaches and Challenges

**Figure 2:**
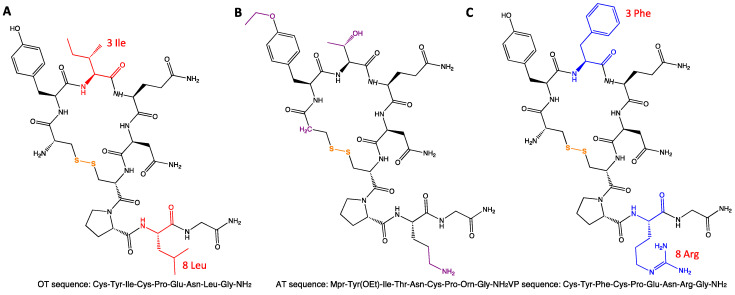
Structure of oxytocin (**A**), atosiban (**B**), and arginine vasopressin (**C**). The main amino acid differences among OT, AT, and VP are labelled in different colours, and the disulphide bond in the peptides is labelled in orange. OT, oxytocin; AT, atosiban, VP, arginine vasopressin.

A successful OTR-targeting strategy relies on the development of reliable ligands that can specifically bind to the receptor. Despite the therapeutic potential, there are only four approved drugs targeting OTR mainly used in obstetrics and gynaecology, and all four drugs are peptides (OT, demoxytocin, carbetocin, and AT) [6], suggesting that peptide-based drug development targeting OTR holds certain advantages. AT is a synthetic analogue with the nonapeptide structure of OT (Figure 2B). Multiple clinical studies are currently ongoing evaluating the effects and feasibility of targeting OTR to treat GI disorders by administration of the peptide agonist OT or the peptide antagonist AT. It has been proven that the binding site for the antagonist is formed by transmembrane helices 1, 2, and 7, with a major contribution to binding affinity by the upper part of helix 7 of the OTR receptor. The binding site for the agonist involves the first three extracellular loops of the receptor [113]. This is helpful for a rational design of the agonists and antagonists.

**Figure 3:**
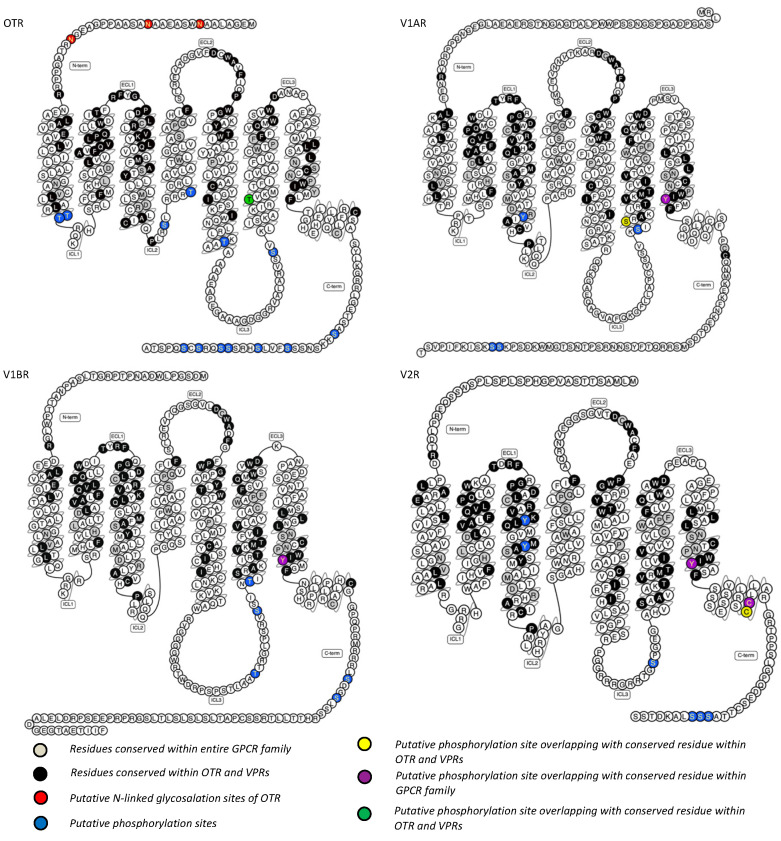
The 2-dimensional diagrams and sequences of human OTR and VPRs, with amino acids presented as single-letter codes. The comparison and similarity between the human oxytocin receptor and the vasopressin receptors are labelled in different colours. OTR, oxytocin receptor; VPRs, vasopressin receptors; V1A/BR, vasopressin receptor 1A/B; V2R, vasopressin receptor 2. The receptor scheme and the putative phosphorylation sites of human VPRs were adapted from the GPCRdb website [118]. The conserved residues were adapted from [18]. The labelled diagram and sequence of human OTR was adapted from our previous publication [119], and its putative phosphorylation sites were adapted from [120].

OT has high similarity to human vasopressin (Figure 2C, VP, also called arginine vasopressin, AVP), with only two amino acid differences at position 3 and position 8 [114]. The human OTR and VPRs share a high degree of sequence similarity (Figure 3). Unsurprisingly, the molecules can cross-react with these receptors and exert their physiological activity, which is not desirable during drug development. Therefore, a major bottleneck in OTR ligand development is the selectivity of the molecules for the specific receptor among the OT/VP receptor family. Fortunately, the recent characterisation of the OTR structure, with the small molecule antagonist retosiban bound to an inactive conformation [115] and OT bound to an active conformation [116,117], has provided advantages for the design and identification of new OTR-specific molecules.

Another concern is OT’s short duration of action and difficulties in reaching brain targets for clinical applications. Therefore, exploring advanced drug delivery techniques and the intranasal administration route could be promising strategies to improve OT’s efficacy. Nanocarriers, including nanoparticles, nanoscale, and liposomes, have been shown to have the advantages of targeted drug release, prolonged blood circulation, enhanced synergies, and superior biocompatibility. There are actually intriguing results of intranasal OT administration eliciting central effects in humans [38], although a clear receptor involvement and the pharmacokinetics of intranasal OT have yet to be demonstrated in large randomized controlled trials.

## 5. Conclusions and Perspectives

Despite the tremendous advance in our understanding of OT/OTR signalling in the GI system, its detailed roles and mechanisms have still not been fully explored. Challenges persist due to signalling complexity, interspecies and gender differences, selectivity concerns, and intricacies in peptide drug delivery.

A major obstacle lies in the non-selectivity of OT binding at VPRs, where OT functions as a partial agonist and induces vasopressin-like side effects such as low blood pressure and osmolality imbalance. Moreover, the physiological consequences of long-term OT application in humans are not well characterized. Thus, it is necessary to address these negative sides in clinical trials using OT for the prevention and treatment of GI disorders. In addition, although the endogenous OTR peptide ligand OT is a widely used drug in obstetrics, it is not the ideal drug candidate due to its poor drug-like properties. The short circulation half-life time and low GI stability of OT is another crucial bottleneck for the therapeutic application, hampering its clinical application.

Future research should shift towards achieving the detailed mechanisms of OT/OTR in the GI system, and the development of OTR-specific compounds with satisfactory pharmacokinetic properties. The generation of specific, efficient, and stable OTR ligands with no effects over other receptors should be our focus, which can in turn help to advance our mechanism studies, serving as molecular tools for investigating OTR signalling in GI disorders and beyond.

## References

[1] Kimura T., Tanizawa O., Mori K., Brownstein M.J., Okayama H. (1992). Structure and expression of a human oxytocin receptor. Nature.

[2] Kimura T., Saji F., Nishimori K., Ogita K., Nakamura H., Koyama M., Murata Y. (2003). Molecular regulation of the oxytocin receptor in peripheral organs. J. Mol. Endocrinol..

[3] Strunecka A. (2010). Cellular an Molecular Biology of Autism Spectrum Disorders (CHAPTER 6).

[4] Modi M.E., Young L.J. (2012). The oxytocin system in drug discovery for autism: Animal models and novel therapeutic strategies. Horm. Behav..

[5] Jurek B., Neumann I.D. (2018). The Oxytocin Receptor: From Intracellular Signaling to Behavior. Physiol. Rev..

[6] Perisic M., Woolcock K., Hering A., Mendel H., Muttenthaler M. (2024). Oxytocin and vasopressin signaling in health and disease. Trends Biochem. Sci..

[7] Japundzic-Zigon N., Lozic M., Sarenac O., Murphy D. (2020). Vasopressin & Oxytocin in Control of the Cardiovascular System: An Updated Review. Curr. Neuropharmacol..

[8] Deing V., Roggenkamp D., Kuhnl J., Gruschka A., Stab F., Wenck H., Burkle A., Neufang G. (2013). Oxytocin modulates proliferation and stress responses of human skin cells: Implications for atopic dermatitis. Exp. Dermatol..

[9] Kerem L., Lawson E.A. (2021). The Effects of Oxytocin on Appetite Regulation, Food Intake and Metabolism in Humans. Int. J. Mol. Sci..

[10] Flanagan L.M., Dohanics J., Verbalis J.G., Stricker E.M. (1992). Gastric motility and food intake in rats after lesions of hypothalamic paraventricular nucleus. Am. J. Physiol..

[11] Esplugues J.V., Barrachina M.D., Beltran B., Calatayud S., Whittle B.J., Moncada S. (1996). Inhibition of gastric acid secretion by stress: A protective reflex mediated by cerebral nitric oxide. Proc. Natl. Acad. Sci. USA.

[12] Petring O.U. (1989). The effect of oxytocin on basal and pethidine-induced delayed gastric emptying. Br. J. Clin. Pharmacol..

[13] Welch M.G., Margolis K.G., Li Z., Gershon M.D. (2014). Oxytocin regulates gastrointestinal motility, inflammation, macromolecular permeability, and mucosal maintenance in mice. Am. J. Physiol. Gastrointest. Liver Physiol..

[14] de Araujo A.D., Mobli M., Castro J., Harrington A.M., Vetter I., Dekan Z., Muttenthaler M., Wan J., Lewis R.J., King G.F. (2014). Selenoether oxytocin analogues have analgesic properties in a mouse model of chronic abdominal pain. Nat. Commun..

[15] Cuesta-Marti C., Uhlig F., Muguerza B., Hyland N., Clarke G., Schellekens H. (2023). Microbes, oxytocin and stress: Converging players regulating eating behavior. J. Neuroendocrinol..

[16] Rao V.V., Loffler C., Battey J., Hansmann I. (1992). The human gene for oxytocin-neurophysin I (OXT) is physically mapped to chromosome 20p13 by in situ hybridization. Cytogenet. Cell Genet..

[17] Wang P., Wang S.C., Liu X., Jia S., Wang X., Li T., Yu J., Parpura V., Wang Y.F. (2022). Neural Functions of Hypothalamic Oxytocin and its Regulation. ASN Neuro.

[18] Gimpl G., Fahrenholz F. (2001). The oxytocin receptor system: Structure, function, and regulation. Physiol. Rev..

[19] Okumura T., Nozu T., Ishioh M., Igarashi S., Funayama T., Kumei S., Ohhira M. (2022). Oxytocin acts centrally in the brain to improve leaky gut through the vagus nerve and a cannabinoid signaling in rats. Physiol. Behav..

[20] Wang Y.K.B., de Lartigue G., Page A.J. (2020). Dissecting the Role of Subtypes of Gastrointestinal Vagal Afferents. Front. Physiol..

[21] Llewellyn-Smith I.J., Kellett D.O., Jordan D., Browning K.N., Travagli R.A. (2012). Oxytocin-immunoreactive innervation of identified neurons in the rat dorsal vagal complex. Neurogastroenterol. Motil..

[22] Ohlsson B., Truedsson M., Djerf P., Sundler F. (2006). Oxytocin is expressed throughout the human gastrointestinal tract. Regul. Pept..

[23] Yu Q., Ji R., Gao X., Fu J., Guo W., Song X., Zhao X., Burnstock G., Shi X., He C. (2011). Oxytocin is expressed by both intrinsic sensory and secretomotor neurons in the enteric nervous system of guinea pig. Cell Tissue Res..

[24] Danhof H.A., Lee J., Thapa A., Britton R.A., Di Rienzi S.C. (2023). Microbial stimulation of oxytocin release from the intestinal epithelium via secretin signaling. Gut Microbes.

[25] Monstein H.J., Grahn N., Truedsson M., Ohlsson B. (2004). Oxytocin and oxytocin-receptor mRNA expression in the human gastrointestinal tract: A polymerase chain reaction study. Regul. Pept..

[26] Feng M., Qin J., Wang C., Ye Y., Wang S., Xie D., Wang P.S., Liu C. (2009). Estradiol upregulates the expression of oxytocin receptor in colon in rats. Am. J. Physiol. Endocrinol. Metab..

[27] Welch M.G., Tamir H., Gross K.J., Chen J., Anwar M., Gershon M.D. (2009). Expression and developmental regulation of oxytocin (OT) and oxytocin receptors (OTR) in the enteric nervous system (ENS) and intestinal epithelium. J. Comp. Neurol..

[28] Richmond C.A., Breault D.T. (2010). Regulation of gene expression in the intestinal epithelium. Prog. Mol. Biol. Transl. Sci..

[29] Takeda S., Kuwabara Y., Mizuno M. (1986). Concentrations and origin of oxytocin in breast milk. Endocrinol. Jpn..

[30] Qin J., Feng M., Wang C., Ye Y., Wang P.S., Liu C. (2009). Oxytocin receptor expressed on the smooth muscle mediates the excitatory effect of oxytocin on gastric motility in rats. Neurogastroenterol. Motil..

[31] Greenwood-Van Meerveld B., Johnson A.C., Grundy D. (2017). Gastrointestinal Physiology and Function. Handb. Exp. Pharmacol..

[32] Yang X., Lou J., Shan W., Ding J., Jin Z., Hu Y., Du Q., Liao Q., Xie R., Xu J. (2021). Pathophysiologic Role of Neurotransmitters in Digestive Diseases. Front. Physiol..

[33] Yanagi S., Sato T., Kangawa K., Nakazato M. (2018). The Homeostatic Force of Ghrelin. Cell Metab..

[34] Iovino M., Messana T., Marucci S., Triggiani D., Giagulli V.A., Guastamacchia E., Piazzolla G., De Pergola G., Lisco G., Triggiani V. (2024). The neurohypophyseal hormone oxytocin and eating behaviors: A narrative review. Hormones.

[35] Brown C.H., Bains J.S., Ludwig M., Stern J.E. (2013). Physiological regulation of magnocellular neurosecretory cell activity: Integration of intrinsic, local and afferent mechanisms. J. Neuroendocrinol..

[36] Liu C.M., Spaulding M.O., Rea J.J., Noble E.E., Kanoski S.E. (2021). Oxytocin and Food Intake Control: Neural, Behavioral, and Signaling Mechanisms. Int. J. Mol. Sci..

[37] Erdman S.E. (2021). Oxytocin and the microbiome. Curr. Opin. Endocr. Metab. Res..

[38] Malewska-Kasprzak M., Jowik K., Tyszkiewicz-Nwafor M. (2023). The use of intranasal oxytocin in the treatment of eating disorders. Neuropeptides.

[39] Spetter M.S., Feld G.B., Thienel M., Preissl H., Hege M.A., Hallschmid M. (2018). Oxytocin curbs calorie intake via food-specific increases in the activity of brain areas that process reward and establish cognitive control. Sci. Rep..

[40] Olszewski P.K., Klockars A., Levine A.S. (2016). Oxytocin: A Conditional Anorexigen whose Effects on Appetite Depend on the Physiological, Behavioural and Social Contexts. J. Neuroendocrinol..

[41] Ohlsson B., Bjorgell O., Ekberg O., Darwiche G. (2006). The oxytocin/vasopressin receptor antagonist atosiban delays the gastric emptying of a semisolid meal compared to saline in human. BMC Gastroenterol..

[42] Maguire S., O’Dell A., Touyz L., Russell J. (2013). Oxytocin and anorexia nervosa: A review of the emerging literature. Eur. Eat. Disord. Rev..

[43] Maguire S., Kesby A., Brownlow R., Hunt G.E., Kim M., McAulay C., Grisham J.R., McGregor I.S., Suraev A., Kevin R.C. (2024). A phase II randomised controlled trial of intranasal oxytocin in anorexia nervosa. Psychoneuroendocrinology.

[44] Borg J., Melander O., Johansson L., Uvnas-Moberg K., Rehfeld J.F., Ohlsson B. (2009). Gastroparesis is associated with oxytocin deficiency, oesophageal dysmotility with hyperCCKemia, and autonomic neuropathy with hypergastrinemia. BMC Gastroenterol..

[45] Hashmonai M., Torem S., Argov S., Barzilai A., Schramek A. (1979). Prolonged post-vagotomy gastric atony treated by oxytocin. Br. J. Surg..

[46] Yoshimoto S., Cerjak D., Babygirija R., Bulbul M., Ludwig K., Takahashi T. (2012). Hypothalamic circuit regulating colonic transit following chronic stress in rats. Stress.

[47] Yang X., Xi T.F., Li Y.X., Wang H.H., Qin Y., Zhang J.P., Cai W.T., Huang M.T., Shen J.Q., Fan X.M. (2014). Oxytocin decreases colonic motility of cold water stressed rats via oxytocin receptors. World J. Gastroenterol..

[48] Wu C.L., Hung C.R., Chang F.Y., Pau K.Y., Wang P.S. (2003). Pharmacological effects of oxytocin on gastric emptying and intestinal transit of a non-nutritive liquid meal in female rats. Naunyn Schmiedebergs Arch. Pharmacol..

[49] Rogers R.C., Hermann G.E. (1987). Oxytocin, oxytocin antagonist, TRH, and hypothalamic paraventricular nucleus stimulation effects on gastric motility. Peptides.

[50] Holmes G.M., Browning K.N., Babic T., Fortna S.R., Coleman F.H., Travagli R.A. (2013). Vagal afferent fibres determine the oxytocin-induced modulation of gastric tone. J. Physiol..

[51] Ohlsson B., Forsling M.L., Rehfeld J.F., Sjolund K. (2002). Cholecystokinin stimulation leads to increased oxytocin secretion in women. Eur. J. Surg..

[52] Babygirija R., Zheng J., Ludwig K., Takahashi T. (2010). Central oxytocin is involved in restoring impaired gastric motility following chronic repeated stress in mice. Am. J. Physiol. Regul. Integr. Comp. Physiol..

[53] Li S., Shi Y., Zhu J., Li J., Wang S., Liu C. (2024). Protective effect of oxytocin on vincristine-induced gastrointestinal dysmotility in mice. Front. Pharmacol..

[54] Xie D.P., Chen L.B., Liu C.Y., Liu J.Z., Liu K.J. (2003). Effect of oxytocin on contraction of rabbit proximal colon in vitro. World J. Gastroenterol..

[55] Xie D., Chen L., Liu C., Liu K. (2006). The inhibitory effects of oxytocin on distal colonic contractile activity in rabbits are enhanced by ovarian steroids. Acta Physiol.

[56] Duridanova D.B., Nedelcheva M.D., Gagov H.S. (1997). Oxytocin-induced changes in single cell K^+^ currents and smooth muscle contraction of guinea-pig gastric antrum. Eur. J. Endocrinol..

[57] Tache Y., Garrick T., Raybould H. (1990). Central nervous system action of peptides to influence gastrointestinal motor function. Gastroenterology.

[58] Flanagan L.M., Olson B.R., Sved A.F., Verbalis J.G., Stricker E.M. (1992). Gastric motility in conscious rats given oxytocin and an oxytocin antagonist centrally. Brain Res..

[59] Rogers R.C., Hermann G.E. (1985). Dorsal medullary oxytocin, vasopressin, oxytocin antagonist, and TRH effects on gastric acid secretion and heart rate. Peptides.

[60] Katoh H., Ohtake M., Sakaguchi T. (1991). Secretion of gastric acid inhibited by oxytocin injected into the hypothalamic paraventricular nucleus in the rat. Neuropeptides.

[61] Asad M., Shewade D.G., Koumaravelou K., Abraham B.K., Vasu S., Ramaswamy S. (2001). Effect of centrally administered oxytocin on gastric and duodenal ulcers in rats. Acta Pharmacol. Sin..

[62] Asad M., Shewade D.G., Koumaravelou K., Abraham B.K., Vasu S., Ramaswamy S. (2001). Gastric antisecretory and antiulcer activity of oxytocin in rats and guinea pigs. Life Sci..

[63] Zhang W., Zhang J., Xu M., Zhang Y. (2007). Effect of oxytocin on gastric ischemia-reperfusion injury in rats. Front. Med. China.

[64] Leng H., Zhang X., Wang Q., Luan X., Sun X., Guo F., Gao S., Liu X., Xu L. (2019). Regulation of stress-induced gastric ulcers via central oxytocin and a potential mechanism through the VTA-Nac dopamine pathway. Neurogastroenterol. Motil..

[65] Calatayud S., Quintana E., Esplugues J., Barrachina M.D. (1999). Role of central oxytocin in the inhibition by endotoxin of distension-stimulated gastric acid secretion. Naunyn Schmiedebergs Arch. Pharmacol..

[66] Kojima M., Hosoda H., Date Y., Nakazato M., Matsuo H., Kangawa K. (1999). Ghrelin is a growth-hormone-releasing acylated peptide from stomach. Nature.

[67] Vila G., Riedl M., Resl M., van der Lely A.J., Hofland L.J., Clodi M., Luger A. (2009). Systemic administration of oxytocin reduces basal and lipopolysaccharide-induced ghrelin levels in healthy men. J. Endocrinol..

[68] Iwakura H., Ariyasu H., Hosoda H., Yamada G., Hosoda K., Nakao K., Kangawa K., Akamizu T. (2011). Oxytocin and dopamine stimulate ghrelin secretion by the ghrelin-producing cell line, MGN3-1 in vitro. Endocrinology.

[69] Shibata K., Hosoda H., Kojima M., Kangawa K., Makino Y., Makino I., Kawarabayashi T., Futagami K., Gomita Y. (2004). Regulation of ghrelin secretion during pregnancy and lactation in the rat: Possible involvement of hypothalamus. Peptides.

[70] Rault J.L., Ferrari J., Pluske J.R., Dunshea F.R. (2015). Neonatal oxytocin administration and supplemental milk ameliorate the weaning transition and alter hormonal expression in the gastrointestinal tract in pigs. Domest. Anim. Endocrinol..

[71] Chu J.Y., Lee L.T., Lai C.H., Vaudry H., Chan Y.S., Yung W.H., Chow B.K. (2009). Secretin as a neurohypophysial factor regulating body water homeostasis. Proc. Natl. Acad. Sci. USA.

[72] Velmurugan S., Brunton P.J., Leng G., Russell J.A. (2010). Circulating secretin activates supraoptic nucleus oxytocin and vasopressin neurons via noradrenergic pathways in the rat. Endocrinology.

[73] Loh J.S., Mak W.Q., Tan L.K.S., Ng C.X., Chan H.H., Yeow S.H., Foo J.B., Ong Y.S., How C.W., Khaw K.Y. (2024). Microbiota–gut–brain axis and its therapeutic applications in neurodegenerative diseases. Signal Transduct. Target. Ther..

[74] Erdman S.E. (2023). Brain trust. Compr. Psychoneuroendocrinol..

[75] Varian B.J., Weber K.T., Erdman S.E. (2023). Oxytocin and the microbiome. Compr. Psychoneuroendocrinol.

[76] Dangoor I., Stanic D., Reshef L., Pesic V., Gophna U. (2021). Specific Changes in the Mammalian Gut Microbiome as a Biomarker for Oxytocin-Induced Behavioral Changes. Microorganisms.

[77] Kingsbury M.A., Bilbo S.D. (2019). The inflammatory event of birth: How oxytocin signaling may guide the development of the brain and gastrointestinal system. Front. Neuroendocrinol..

[78] Poutahidis T., Kearney S.M., Levkovich T., Qi P., Varian B.J., Lakritz J.R., Ibrahim Y.M., Chatzigiagkos A., Alm E.J., Erdman S.E. (2013). Microbial symbionts accelerate wound healing via the neuropeptide hormone oxytocin. PloS ONE.

[79] Bravo J.A., Forsythe P., Chew M.V., Escaravage E., Savignac H.M., Dinan T.G., Bienenstock J., Cryan J.F. (2011). Ingestion of Lactobacillus strain regulates emotional behavior and central GABA receptor expression in a mouse via the vagus nerve. Proc. Natl. Acad. Sci. USA.

[80] Rash J.A., Aguirre-Camacho A., Campbell T.S. (2014). Oxytocin and pain: A systematic review and synthesis of findings. Clin. J. Pain..

[81] Boll S., Almeida de Minas A.C., Raftogianni A., Herpertz S.C., Grinevich V. (2018). Oxytocin and Pain Perception: From Animal Models to Human Research. Neuroscience.

[82] Lopes S., Osório F.L. (2023). Effects of intranasal oxytocin on pain perception among human subjects: A systematic literature review and meta-analysis. Horm. Behav..

[83] Drewes A.M., Olesen A.E., Farmer A.D., Szigethy E., Rebours V., Olesen S.S. (2020). Gastrointestinal pain. Nat. Rev. Dis. Primers.

[84] Xu S., Qin B., Shi A., Zhao J., Guo X., Dong L. (2018). Oxytocin inhibited stress induced visceral hypersensitivity, enteric glial cells activation, and release of proinflammatory cytokines in maternal separated rats. Eur. J. Pharmacol..

[85] Tsushima H., Zhang Y., Muratsubaki T., Kanazawa M., Fukudo S. (2021). Oxytocin antagonist induced visceral pain and corticotropin-releasing hormone neuronal activation in the central nucleus of the amygdala during colorectal distention in mice. Neurosci. Res..

[86] Alfvén G. (2004). Plasma oxytocin in children with recurrent abdominal pain. J. Pediatr. Gastroenterol. Nutr..

[87] Madrazo I., Franco-Bourland R.E., Leon-Meza V.M., Mena I. (1987). Intraventricular somatostatin-14, arginine vasopressin, and oxytocin: Analgesic effect in a patient with intractable cancer pain. Appl. Neurophysiol..

[88] Ohlsson B., Truedsson M., Bengtsson M., Torstenson R., Sjolund K., Bjornsson E.S., Simren M. (2005). Effects of long-term treatment with oxytocin in chronic constipation; a double blind, placebo-controlled pilot trial. Neurogastroenterol. Motil..

[89] Louvel D., Delvaux M., Felez A., Fioramonti J., Bueno L., Lazorthes Y., Frexinos J. (1996). Oxytocin increases thresholds of colonic visceral perception in patients with irritable bowel syndrome. Gut.

[90] Okumura T., Nozu T., Kumei S., Ohhira M. (2019). Central oxytocin signaling mediates the central orexin-induced visceral antinociception through the opioid system in conscious rats. Physiol. Behav..

[91] Iseri S.O., Gedik I.E., Erzik C., Uslu B., Arbak S., Gedik N., Yegen B.C. (2008). Oxytocin ameliorates skin damage and oxidant gastric injury in rats with thermal trauma. Burns.

[92] Chen D., Zhao J., Wang H., An N., Zhou Y., Fan J., Luo J., Su W., Liu C., Li J. (2015). Oxytocin evokes a pulsatile PGE2 release from ileum mucosa and is required for repair of intestinal epithelium after injury. Sci. Rep..

[93] Klein B.Y., Tamir H., Hirschberg D.L., Ludwig R.J., Glickstein S.B., Myers M.M., Welch M.G. (2016). Oxytocin opposes effects of bacterial endotoxin on ER-stress signaling in Caco2BB gut cells. Biochim. Biophys. Acta.

[94] Chukwunyere U., Mercan M., Sehirli A.O., Abacioglu N. (2022). Possible cytoprotective mechanisms of oxytocin against 5-fluorouracil-induced gastrointestinal mucositis. Mol. Biol. Rep..

[95] Marchese S.H., Naftaly J.P., Pandolfino J. (2024). Acceptance and commitment therapy for the treatment of irritable bowel syndrome and inflammatory bowel disease: A narrative review. Transl. Gastroenterol. Hepatol..

[96] Quiros M. (2020). Therapeutic Opportunities for Repair GPCRs during Intestinal Mucosal Wound Healing. Trends Mol. Med..

[97] Ishioh M., Nozu T., Okumura T. (2024). Brain Neuropeptides, Neuroinflammation, and Irritable Bowel Syndrome. Digestion.

[98] Li J., Liu H., Guo F., Guo R., Zhang H., He X., Ming X., Ma X., Shang G., Ji P. (2023). Increased GABAergic projections in the paraventricular nucleus regulate colonic hypersensitivity via oxytocin in a rat model of irritable bowel syndrome. Neuroreport.

[99] Li T., Wang P., Wang S.C., Wang Y.F. (2016). Approaches Mediating Oxytocin Regulation of the Immune System. Front. Immunol..

[100] Jiang J., Yang M., Tian M., Chen Z., Xiao L., Gong Y. (2023). Intertwined associations between oxytocin, immune system and major depressive disorder. Biomed. Pharmacother..

[101] Wang X., Chen D., Guo M., Ning Y., Geng M., Guo J., Gao J., Zhao D., Zhang Y., Li Q. (2024). Oxytocin Alleviates Colitis and Colitis-Associated Colorectal Tumorigenesis via Noncanonical Fucosylation. Research.

[102] Tang Y., Shi Y., Gao Y., Xu X., Han T., Li J., Liu C. (2019). Oxytocin system alleviates intestinal inflammation by regulating macrophages polarization in experimental colitis. Clin. Sci..

[103] Dou D., Liang J., Zhai X., Li G., Wang H., Han L., Lin L., Ren Y., Liu S., Liu C. (2021). Oxytocin signalling in dendritic cells regulates immune tolerance in the intestine and alleviates DSS-induced colitis. Clin. Sci..

[104] Işeri S.O., Sener G., Sağlam B., Gedik N., Ercan F., Yeğen B.C. (2005). Oxytocin ameliorates oxidative colonic inflammation by a neutrophil-dependent mechanism. Peptides.

[105] Işeri S.O., Sener G., Saglam B., Gedik N., Ercan F., Yegen B.C. (2005). Oxytocin protects against sepsis-induced multiple organ damage: Role of neutrophils. J. Surg. Res..

[106] Yu Y., Li J., Liu C. (2022). Oxytocin suppresses epithelial cell-derived cytokines production and alleviates intestinal inflammation in food allergy. Biochem. Pharmacol..

[107] Lerman B., Harricharran T., Ogunwobi O.O. (2018). Oxytocin and cancer: An emerging link. World J. Clin. Oncol..

[108] Carr J.C., Sherman S.K., Wang D., Dahdaleh F.S., Bellizzi A.M., O’Dorisio M.S., O’Dorisio T.M., Howe J.R. (2013). Overexpression of membrane proteins in primary and metastatic gastrointestinal neuroendocrine tumors. Ann. Surg. Oncol..

[109] Sun J., Xu Z., Mao Y., Zhang T., Qin Y., Hua D. (2021). Prognostic role of oxytocin receptor in colon adenocarcinoma. Open Med..

[110] Sherman S.K., Carr J.C., Wang D., O’Dorisio M.S., O’Dorisio T.M., Howe J.R. (2013). Gastric inhibitory polypeptide receptor (GIPR) is a promising target for imaging and therapy in neuroendocrine tumors. Surgery.

[111] Harricharran T., Ogunwobi O.O. (2020). Oxytocin and oxytocin receptor alterations, decreased survival, and increased chemoresistance in patients with pancreatic cancer. Hepatobiliary Pancreat. Dis. Int..

[112] Wang M., Guo X., Yang M., Zhang Y., Meng F., Chen Y., Chen M., Qiu T., Li J., Li Z. (2022). Synergistic antitumor activity of 5-fluorouracil and atosiban against microsatellite stable colorectal cancer through restoring GATA3. Biochem. Pharmacol..

[113] Postina R., Kojro E., Fahrenholz F. (1996). Separate agonist and peptide antagonist binding sites of the oxytocin receptor defined by their transfer into the V2 vasopressin receptor. J. Biol. Chem..

[114] Nashar P.E., Whitfield A.A., Mikusek J., Reekie T.A. (2022). The Current Status of Drug Discovery for the Oxytocin Receptor. Methods Mol. Biol..

[115] Waltenspühl Y., Schöppe J., Ehrenmann J., Kummer L., Plückthun A. (2020). Crystal structure of the human oxytocin receptor. Sci. Adv..

[116] Meyerowitz J.G., Robertson M.J., Barros-Álvarez X., Panova O., Nwokonko R.M., Gao Y., Skiniotis G. (2022). The oxytocin signaling complex reveals a molecular switch for cation dependence. Nat. Struct. Mol. Biol..

[117] Waltenspühl Y., Ehrenmann J., Vacca S., Thom C., Medalia O., Plückthun A. (2022). Structural basis for the activation and ligand recognition of the human oxytocin receptor. Nat. Commun..

[118] Pandy-Szekeres G., Munk C., Tsonkov T.M., Mordalski S., Harpsoe K., Hauser A.S., Bojarski A.J., Gloriam D.E. (2018). GPCRdb in 2018: Adding GPCR structure models and ligands. Nucleic Acids Res..

[119] Liu H., Gruber C.W., Alewood P.F., Möller A., Muttenthaler M. (2020). The oxytocin receptor signalling system and breast cancer: A critical review. Oncogene.

[120] Plested C.P., Bernal A.L. (2001). Desensitisation of the oxytocin receptor and other G-protein coupled receptors in the human myometrium. Exp Physiol..

